# Mini Transsternal Approach to the Anterior High Thoracic Spine (T1–T4 Vertebrae)

**DOI:** 10.1155/2016/4854217

**Published:** 2016-04-27

**Authors:** Christian Brogna, Bhaskar Thakur, Leslie Fiengo, Sandra Maria Tsoti, Alessandro Landi, Giulio Anichini, Francesco Vergani, Irfan Malik

**Affiliations:** ^1^Department of Neurosurgery, King's College Hospital NHS Foundation Trust, London, UK; ^2^Department of Vascular Surgery, King's College Hospital NHS Foundation Trust, London, UK; ^3^Faculty of Medicine, Imperial College London, London, UK; ^4^Department of Neurosurgery, “Sapienza” University of Rome, Rome, Italy

## Abstract

*Purpose*. The anterior high thoracic spine is one of the most complex segments to be accessed surgically due to anatomical constraints and transitional characteristics. We describe in detail the mini transsternal approach to metastatic, infective, traumatic, and degenerative pathologies of T1 to T4 vertebral bodies. We analyse our surgical series, indications, and outcomes.* Methods*. Over a 5-year period 18 consecutive patients with thoracic myelopathy due to metastatic, infective, traumatic, and degenerative pathologies with T1 to T4 vertebral bodies involvement received a mini transsternal approach with intraoperative monitoring. Frankel scoring system was used to grade the neurological status.* Results*. Mean follow-up was 40 months. 78% patients improved in Frankel grade after surgery and 22% patients remained unchanged. Average operation time was 210 minutes. There were no intraoperative complications. One patient developed postoperative pneumonia successfully treated with antibiotics.* Conclusion*. The mini transsternal is a safe approach for infective, metastatic, traumatic, and degenerative lesions affecting the anterior high thoracic spine and the only one allowing an early and direct visualisation of the anterior theca. This approach overcomes the anatomical constraints of this region and provides adequate room for optimal reconstruction and preservation of spinal alignment in the cervicothoracic transition zone with good functional patient outcomes.

## 1. Introduction

Neoplastic lesions, infectious processes, disc herniations, traumatic fractures, and severe kyphotic deformities can occur in the anterior upper thoracic spine [1–9]. Historically, anterior access to the cervicothoracic junction down to T4 vertebrae has been hampered by anatomical constraints, namely, the supra-aortic trunks, and the transition from cervical lordosis to thoracic kyphosis [8–15]. Adequate and early visualisation of the anterior thecal sac and the need to avoid postoperative kyphotic deformities and instability are paramount [8, 9, 14–18].

We describe our experience with the mini transsternal approach, in a wide range of diseases involving the anterior upper thoracic spine. This approach provides excellent direct access to the upper thoracic vertebral bodies, optimising both the neurological outcome and the preservation of spinal alignment in the cervicothoracic transition zone.

## 2. Materials and Methods

### 2.1. Patient Population

Between January 2010 and January 2015, 18 patients with various pathological entities involving the anterior upper thoracic spine from T1 to T4 received a mini transsternal approach by the most senior author I. Malik (Table 1).

All patients presented with thoracic myelopathy, and the average time to diagnosis was 5 months (1–9 months). Neurological status was graded according to Frankel scoring system. Preoperative neuroimaging included MRI spine and CT scan with 3D reconstructions. Intraoperative neuromonitoring with SSEPs and MEPs was used in all cases.

### 2.2. Mini Transsternal Approach: Operative Technique

**Figure 1:**
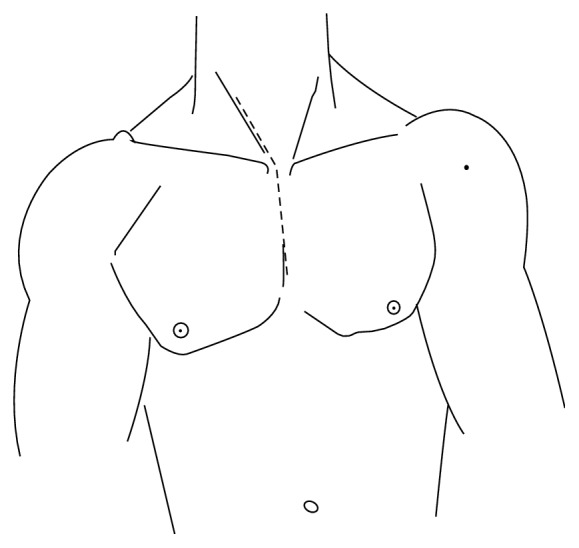
Vertical incision in the midline of the upper sternum, prolonged cranially in the cervical region along the anteromedial border of the right sternocleidomastoid muscle.

**Figure 2:**
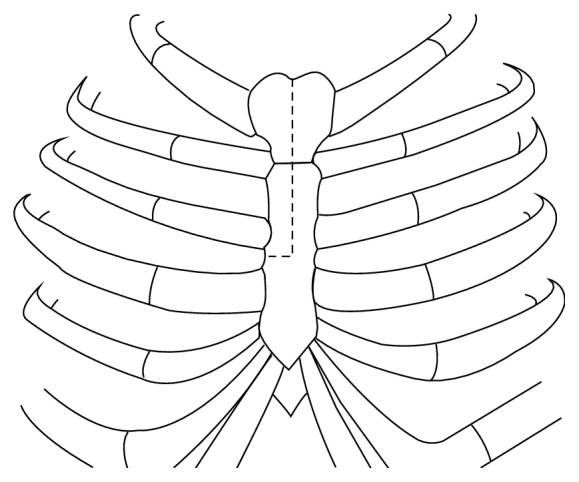
Inverted T shaped ministernotomy extended caudally to the third rib.

**Figure 3:**
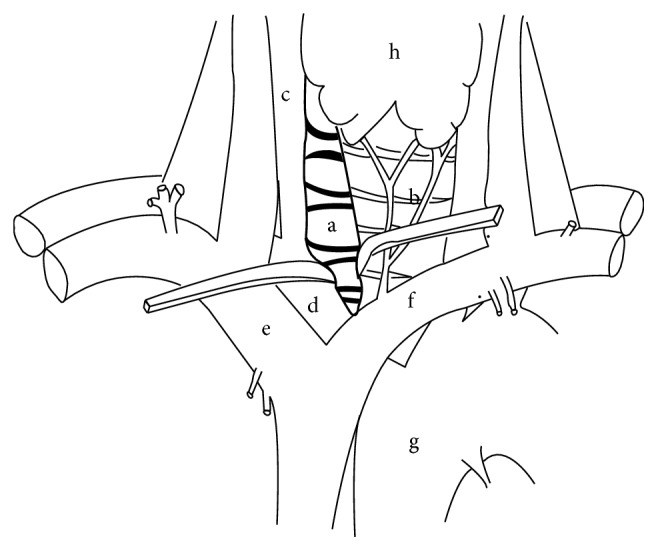
(a) Spine. (b) Trachea. (c) Right common carotid artery. (d) Right brachiocephalic artery. (e) Right innominate vein. (f) Left innominate vein. (g) Aorta. (h) Thyroid gland.

Patients receiving endotracheal general anaesthesia and neuromonitoring (SSEPs and MEPs) were placed supine with the neck slightly extended. A vertical incision was made in the midline of the upper sternum and prolonged cranially in the cervical region along the anteromedial border of the right sternocleidomastoid muscle (Figure 1). The platysma was divided, precervical and pretracheal fascia were opened, and the sternal part of the sternocleidomastoid muscle was divided and dissected away from deeper structures. The upper part of the manubrium was cleared from the insertion of the subhyoid muscles. An inverted T shaped ministernotomy extended caudally to the third rib was performed and a rib retractor was placed (Figure 2). A plane was identified and dissected between the oesophagus and trachea on one side and the carotid sheath on the other side. In particular, access to prevertebral space was gained through an inner window of the brachiocephalic artery, whose borders are the tracheoesophageal sheath medially and the brachiocephalic and the common carotid artery laterally (Figure 3). Vertebral bodies down to T4 could be generously exposed according to the level of interest. In two cases, where a caudal extension of the approach was necessary, the “outside window of the brachiocephalic artery” (basal: left innominate vein; left: brachiocephalic artery; right: proximal portion of the right innominate vein) was used to gain access to the upper T5 [16]. In this case, the trachea, oesophagus, and brachiocephalic artery were retracted to the left, whereas the proximal portion of the right innominate vein is retracted to the right and the left innominate vein inferolaterally. In fact, this approach wards off the limitation of the aortic arch and its branches. Inferior thyroid artery and veins can be ligated for better exposure.

Once exposure of the anterior body of the thoracic vertebrae is achieved due to the difficulty of obtaining intraoperative radiographs at this level, it may be necessary to mark a disc of the lower cervical spine and count down. It is worthwhile to get an estimate of the midline and lateral boundaries by first identifying these landmarks in normal segments adjacent to the area of pathology.

The operating microscope is then introduced. Standard microsurgical spinal instruments were used. The disc is incised with a number 11 scalpel laterally to identify the Luschka joints and then removed to expose the posterior longitudinal ligament (PLL). When vertebrectomy is planned, the discectomy above and below each vertebra prior to the corpectomy allows an early identification of the dura.

The corpectomy is performed in a rectangular configuration to ensure adequate decompression and create an excellent graft recipient site. PLL is then removed piecemeal and adequacy of decompression is judged by carefully sliding the blunt nerve hook under the superior and inferior vertebral bodies. Removal of the cartilaginous endplate while preserving the subchondral cortical bone and ensuring that endplates are parallel are paramount for adequate fusion to occur.

The kyphotic curvature of the cervicothoracic junction also poses a problem with the anterior plates. Anterior plates with minimal bending are used. Length of screws should be estimated by preoperative CT, since the rostrocaudal angulation of the screws and the posterior vertebral cortex cannot be visualised on fluoroscopy.

The wound is closed over Kieselgel drains. The sternum was carefully closed with steel wires to avoid pseudarthrosis of the sternum, which would require another intervention. The soft tissue anatomy is then restored and skin closed.

## 3. Results

There were no intraoperative complications recorded. The average operation time was 210 minutes (150–280 minutes). Intraoperative bleeding was 450–1100 mL (average 800 mL), which was related to the treated pathology rather than to the approach. In fact a minor loss of blood always less than 150 mL was registered in all cases while performing the approach phase. Moreover, we never felt a standard transsternal approach would have offered more room to visualise the anterior cervicothoracic vertebral junction in any case, due to the fact that the lower extension of the approach is hampered by the great vessels rather than the caudal extension of the sternal opening. We did not encounter any dural tear or cerebrospinal fluid leak.

One patient had postoperative pneumonia that was treated successfully with antibiotic therapy. All patients demonstrated good pain control in the postoperative period and only two patients experienced intercostal pain, which resolved with intercostal nerve blocks. Of interest, none of the patients experienced any neurological deterioration after surgery. None of the patients had wound complications.

Follow-up was obtained in all patients, with a mean follow-up duration of 40 ± 24 months (range 6–77 months). At the last follow-up, 9 (75%) of the 12 patients with Frankel grade D improved to grade E; 3 (75%) of the 4 patients with Frankel grade C improved to grade D or E and, of the 2 patients with Frankel grade B, one improved to grade C and the other to grade D.

Despite the fact that 4 patients (22%) did not improve in Frankel grade after surgery, they were satisfied with the results of the operations as no further neurological deterioration had occurred and they experienced relief of their thoracic back pain.

Serial radiographs and CT scans were obtained in each case to confirm stability of instrumentation. No measurable change in spinal alignment was noted between the early postoperative status and follow-up. None of the patients showed any sign of instrument migration or failure.

### 3.1. Case Illustration 1: Disc Herniation (Figures 4(a)–4(c))

A 33-year-old male presented with a 5-month history of progressive paralysis of the lower extremities, midback pain, and myelopathic signs with decreased sensation below T5 distribution. CT and MRI revealed a T3/4 central disc herniation with severe spinal cord compression. The calcified herniated disc occupied almost the entire spinal canal. The patient underwent a mini transsternal approach and T3/4 decompression and fusion with a prefilled cage. Postoperatively, the patient experienced relief of his symptoms and began to walk by himself on the 7th day postoperatively. His Frankel grade improved from D to E. The patient returned to work 4 months after the operation, reporting complete pain relief with no axial back pain.

### 3.2. Case Illustration 2: Spine Metastases (Figures 5(a)–5(c))

A 55-year-old female with a 6-month history of interscapular pain and bilateral upper limb paresthesia and 2-week history of lower limb weakness was admitted with urinary retention requiring urgent catheterisation. She had no significant medical history. On examination, she was quadriparetic (grip 3/5, right lower limb 3/5, and left lower limb 1/5) with a T2 sensory level. She was able to feel the catheter. A 3 cm hard right breast lump was felt. MRI showed T1 vertebral collapse with cord compression and disease infiltrating C7 and T2 with further deposits throughout the thoracolumbar spine. CT scan showed a 3 cm right breast lump with enlarged axillary lymph nodes. She received a mini transsternal approach and T2-T3 corpectomies. A bone graft 5 cm in length from the iliac crest was inserted in the trench, and an anterior spinal titanium plate was placed between T1 and T4 for stabilization. She also had a posterior cervicothoracic fixation with pedicle screws in a second stage a week later. Histopathology exam revealed a metastatic breast carcinoma. At 6-month follow-up no further neurological deterioration was noticed and her Frankel grade C remained unchanged.

### 3.3. Case Illustration 3: Kyphotic Deformity in Ankylosing Spondylitis (Figures 6(a)–6(c))

A 39-year-old female with ankylosing spondylitis and previous C5/6 anterior cervical fixation and fusion presented with a progressive flexion deformity of the neck and weakness of the arms and legs. CT scan showed a C6/7 subluxation. The patient had an extensive anterior cervical fixation via a mini transsternal approach, removal of previous C5/6 fixation, C6/7 discectomy with iliac crest bone graft, and insertion of a C5-T2 anterior plate and application of a cervical halo. At last follow-up her Frankel grade D remained unchanged.

### 3.4. Case Illustration 4: Spine Tuberculosis (Figures 7(a)–7(c) and 8(a)-8(b))

A 26-year-old female student presented with 5-month history of upper back pain and 4-day weakness in the arms and legs with numbness from the chest down. Neurologically she had decreased sensation below T7, grade 4/5 power in the upper limbs, 3/5 power in the lower limbs with brisk reflexes, and increased tone and clonus. Weight loss in the past few months was noted, but there was no fever or productive cough. She was not immunised for tuberculosis and did not have family history positive for tuberculosis. MRI showed a paraspinal abscess from C7-T1, with collapse of the vertebral body and cervical cord compression. A mini transsternal approach, anterior C7-T1 vertebrectomy with decompression of the ventral sac, was performed. Reconstruction was achieved with a mesh, iliac bone graft, and an anterior plate. A week later patient received a C5-T4 posterior fixation for stability, which allowed early mobilisation of the patient. At 1-year follow-up her Frankel grade improved to grade E.

## 4. Discussion

The value of the anterior approach to the spine in providing greater access and better outcomes to lesions affecting the vertebral body has been amply demonstrated. However, anterior access to vertebral bodies from T1 to T4 is probably the most challenging due to several anatomical constraints [7, 9, 11–15, 19].

Historically, most pioneering surgical efforts were directed to the treatment of Pott's disease [6, 7, 20], but similar principles were then applied to the management of primary and metastatic tumors and pathological fracture-dislocation resulting in direct posterior displacement of bone fragments, disc herniations, and severe kyphotic deformities with anterior cord compression [1–9, 21].

In 1894 Mirnard resected a portion of rib and transverse process (costotransversectomy) to have a limited access to the vertebral bodies [6]. In 1954 Capener modified this approach resecting a longer segment of the rib to allow an anterolateral decompression of the spinal cord [22].

In 1957 Cauchoix and Binet finally described a direct approach to the cervicothoracic region through a median sternotomy [23]. Unfortunately, since Hodgson et al. in 1960 [4] published the use of a direct anterior transsternal approach in a series of 10 cases with an operative mortality of 40%, advocating abandoning direct anterior exposure in favour of thoracotomy and resection of the first rib, the transsternal approach was abandoned for more than two decades. Then Sundaresan et al. in 1984 [7] demonstrated the technical feasibility of a direct surgical approach to the upper thoracic vertebrae by partial resection of the manubrium and clavicle and showed the low morbidity rate associated with the technique. “The postoperative course in all patients was relatively benign and similar to that in patients undergoing surgery for disc excision by the methods of Cloward or Robinson and Smith.”

Posterior, posterolateral, and anterolateral exposures only allow for indirect decompression of the ventral aspect of the spine, thus increasing the risk of neurological injury. In particular thoracic lateral approaches to T1 to T4 vertebrae require elevation of the scapula with extensile muscle dissection and rib resection, leading to significant morbidity [24]. A cervical approach does not allow good spinal cord decompression below T1 and a good osteosynthesis due to the obliquity of the access [21].

On the other hand, anterior approaches provide decompression with a direct view of the compressive elements. This ensures that neural elements are not compromised during decompression. Moreover, wide decompression can be readily addressed with options for grafting and fixation.

The transcervical supraclavicular approach provides access to this region of the spine without disrupting the sternum but carries some disadvantages. The approach is limited caudally to T3 and the operative field becomes steep for second and third thoracic vertebrae over the manubrium limiting the surgeon ability to place anterior instrumentation [11].

Complete sternotomy is not necessary because the heart and great vessels limit the caudal extent of the exposure. The transmanubrial transclavicular approach provides a direct route down to T3 vertebra and also provides autologous bone graft and obviates the need to harvest bone from a second site. However, the surgical field is limited by the extent of manubrial resection, which can result in a large manubrial defect and the impossibility to access T4 vertebrae. More recently Xiao et al. described a modified transmanubrial approach without resection of the clavicle in which sternotomy is performed up to 2 cm below the sternal angle. Therefore it cannot be considered a pure transmanubrial approach, since a partial sternotomy is required. With this approach and going through the right space of the brachiocephalic trunk, T3 to T5 can be reached with a good working angle [16].

Our series demonstrates the technical feasibility of a direct surgical approach to the upper thoracic vertebrae with a mini transsternal approach and shows the low morbidity rate associated with the technique. 75% of our patients experienced a substantial neurological improvement upgrading in the Frankel scale. Of notice, none of the patients suffered from any neurological postoperative deterioration. This is mainly due to the direct surgical view of the anterior portion of the theca allowing early and direct visualisation of the dural sac. Moreover, as demonstrated in our series, reconstruction of the cervicothoracic junction could be easily performed by means of the direct access to the anterior column of the spine, providing immediate stability with instrumentation and as a consequence preserving neurological integrity and pain relief, maintaining alignment without deformity, and providing early mobilisation. In patients with disc herniations, discectomy was followed by the insertion of a cage following the same principles adopted for anterior approach to the cervical spine.

We believe that indications for the mini transsternal approach to the high thoracic spine are (1) primary or metastatic tumors confined to the vertebral body or when an anterior decompression is required as an adjunct to a posterior decompression; (2) central or centrolateral calcified symptomatic disc herniations; (3) pathological fracture-dislocation resulting in direct posterior displacement of bone fragments; (4) infectious diseases like tuberculosis, with involvement of the anterior column and direct ventral compression on the thecal sac; and (5) severe kyphotic deformities with anterior spinal cord compression.

## 5. Conclusions

The mini transsternal is a safe approach for infective, metastatic, traumatic, and degenerative lesions affecting the anterior high thoracic spine and the only one allowing an early and direct visualisation of the anterior theca. This approach overcomes the anatomical constraints of this region and provides adequate room for optimal reconstruction and preservation of spinal alignment in the cervicothoracic transition zone with good functional patient's outcomes.

## Figures and Tables

**Figure 4:**
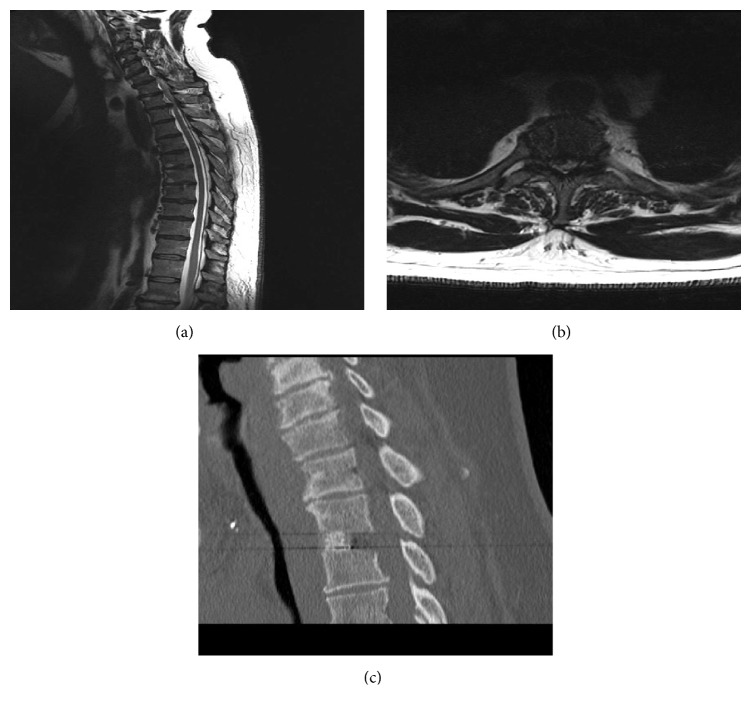
(a) Case illustration 1: sagittal T2-weighted MRI showing a T3/4 central calcified disc herniation with cord compression. (b) Case illustration 1: axial T2-weighted MRI showing a T3/4 central calcified disc herniation with cord compression. (c) Case illustration 1: postoperative CT scan after a mini transsternal approach showing level of discectomy and fusion. Patient's Frankel grade improved from D to E and returned to work 4 months after the operation, reporting complete pain relief with no axial back pain.

**Figure 5:**
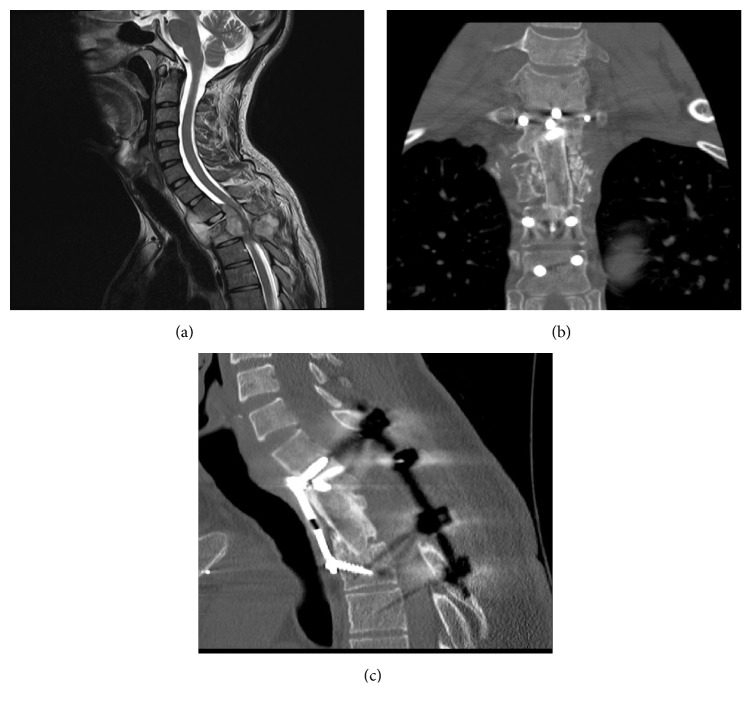
(a) Case illustration 2: T2-weighted MRI showing a T2-3 neoplastic lesion associated with spinal cord compression. (b) Case illustration 2: postoperative CT scan showing the titanium construct with iliac bone autograft and adequate decompression of the spinal canal. (c) Case illustration 2: postoperative CT scan showing the titanium construct with iliac bone autograft and adequate decompression of the spinal canal. Patient's Frankel grade improved from C to E.

**Figure 6:**
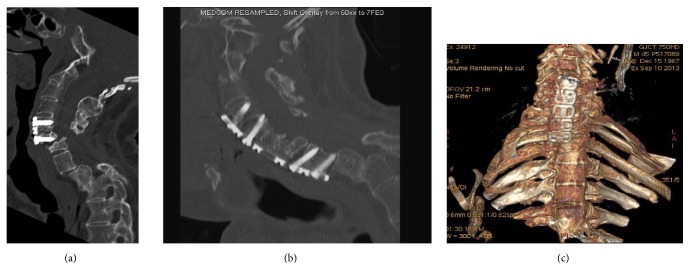
(a) Case illustration 3: preoperative CT showing C6-C7 subluxation with spinal canal compromise and previous C5-C6 fixation in the context of ankylosing spondylitis. (b) Case illustration 3: postop CT scan showing the correction of the spine alignment at the cervicothoracic junction, the widened spinal canal, and the C5-T2 fixation. (c) Case illustration 3: postop CT scan with 3D reconstruction showing the correction of the spine alignment at the cervicothoracic junction. Patient remained neurologically stable and did not experience any further neurological deterioration.

**Figure 7:**
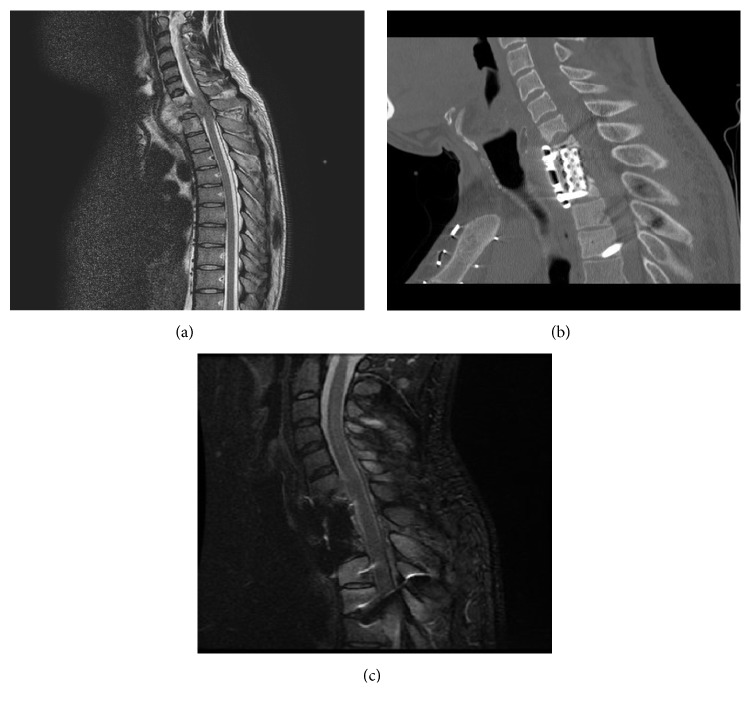
(a) Case illustration 4: preoperative MRI showing a paraspinal abscess in C7-T1, with collapse of the vertebral body and cervical cord compression. (b) Case illustration 4: postoperative CT scan showing C6-T2 anterior fixation and adequate decompression of the spinal cord. Patient received also a posterior fixation a week later. (c) Case illustration 4: postoperative MRI scan showing C6-T2 anterior fixation and adequate decompression of the spinal cord. Patient received also a posterior fixation a week later. Her Frankel grade improved to grade E at 1-year follow-up.

**Figure 8:**
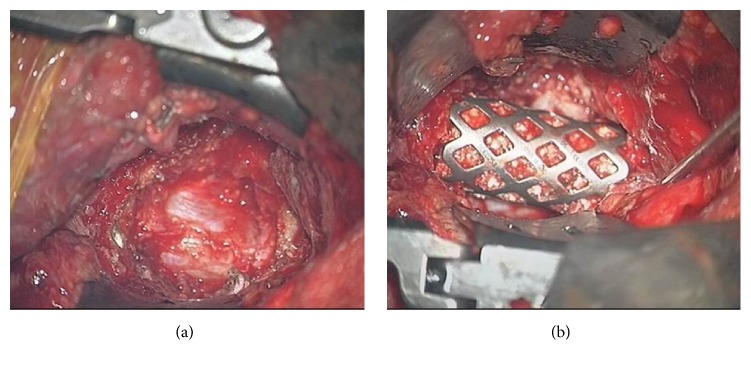
(a) Case illustration 4: intraoperative picture under the microscope showing the ministernotomy and the direct view of the anterior high thoracic vertebral bodies achievable with a mini transsternal approach. (b) Case illustration 4: intraoperative photo of the placement of the mesh filled with autologous iliac bone. The mini transsternal approach allows an excellent route for anterior spine reconstruction.

**Table 1:** Case series.

Case number	Age/sex	Disease	Affected level	Anterior spinal fusion level	Posterior fix	Operative time (min)	Blood loss (mL)	Perioperative complications	Frankel grade Preoperative	Frankel grade Last follow-up
1	33, M	Disc	T3/4	—	No	150	450	—	D	E
2	44, F	Disc	T3/4	—	No	180	700	—	D	E
3	38, F	Disc	T2/3	—	No	170	560	—	C	D
4	39, F	Adjacent level pathology (ankylosing spondylitis)	C6/7 subluxation with severe flexion deformity	C4-T3	Yes	280	900	—	D	D
5	52, F	Metastasis (breast)	T1	C7/T2	Yes	180	750	Pneumonia	D	E
6	46, F	Metastasis (breast)	T1 (C7/T2 corpectomy)	C6-T3	Yes	210	900	—	D	D
7	53, F	Metastasis (breast)	T3	T2/T4	No	200	860	Intercostal pain	C	C
8	50, M	Metastasis (colon)	T2	T1/T3	No	170	650	—	D	D
9	40, M	Metastasis (lung)	T3	T2–T4	No	220	850	—	D	E
10	43, M	Metastasis (leiomyosarcoma)	T2/3	T1/4	No	250	750	—	D	D
11	39, F	Traumatic fracture	T3	T2/T4	Yes	250	950	—	B	D
12	26, M	TB	T1/2	C7/T3	Yes	180	1050	—	D	E
13	43, M	TB	T2/3	T1/T4	No	250	750	—	C	E
14	45, F	TB	T1/2	C7/T3	Yes	230	900	—	C	D
15	53, M	TB	T2/3	T1/T4	No	180	1100	—	B	C
16	46, F	TB	T1/2	C7/T3	No	230	870	Intercostal pain	D	E
17	48, M	TB	T3	T2/T4	No	220	750	—	D	E
18	39	TB	T2/3	T1/T4	No	230	670	—	D	E
